# Immune response pattern in recurrent *Plasmodium vivax* malaria

**DOI:** 10.1186/s12936-016-1501-5

**Published:** 2016-08-31

**Authors:** Yury Oliveira Chaves, Allyson Guimarães da Costa, Marcelo Luís Monteiro Pereira, Marcus Vinícius Guimarães de Lacerda, Jordana Grazziela Coelho-dos-Reis, Olindo Assis Martins-Filho, Andréa Teixeira-Carvalho, Adriana Malheiro, Wuelton Marcelo Monteiro, Patrícia Puccinelli Orlandi, Claudio Romero Farias Marinho, Paulo Afonso Nogueira

**Affiliations:** 1 Instituto Leônidas e Maria Deane, Fundação Oswaldo Cruz (FIOCRUZ), Manaus, AM Brazil; 2 Programa de Pós-Graduação em Medicina Tropical, Universidade do Estado do Amazonas (UEA), Manaus, AM Brazil; 3 Fundação de Medicina Tropical Dr. Heitor Vieira Dourado (FMT-HVD), Manaus, AM Brazil; 4 Departamento de Ensino e Pesquisa, Fundação Hospitalar de Hematologia e Hemoterapia do Amazonas (HEMOAM), Manaus, AM Brazil; 5 Departamento de Parasitologia, Instituto de Ciências Biológicas, Universidade de São Paulo (USP), São Paulo, SP Brazil; 6 Grupo Integrado de Pesquisas em Biomarcadores de Diagnóstico e Monitoração, Centro de Pesquisas René Rachou, Fundação Oswaldo Cruz (FIOCRUZ), Belo Horizonte, MG Brazil; 7 Programa de Pós-Graduação em Imunologia Básica e Aplicada, Universidade Federal do Amazonas (UFAM), Manaus, AM Brazil

**Keywords:** Malaria, Recurrence, *Plasmodium vivax*, Interleukin-10, CD4^+^ T-cells, CD8^+^ T-cells

## Abstract

**Background:**

*Plasmodium vivax* is the causative agent of human malaria of large geographic distribution, with 35 million cases annually. In Brazil, it is the most prevalent species, being responsible by around 70 % of the malaria cases.

**Methods:**

A cross-sectional study was performed in Manaus (Amazonas, Brazil), including 36 adult patients with primary malaria, 19 with recurrent malaria, and 20 endemic controls. The ex vivo phenotypic features of circulating leukocyte subsets (CD4^+^ T-cells, CD8^+^ T-cells, NK, NKT, B, B1 and Treg cells) as well as the plasmatic cytokine profile (IL-2, IL-4, IL-6, IL-10, TNF and IFN-γ) were assessed, aiming at establishing patterns of immune response characteristic of primary malaria vs recurrent malaria as compared to endemic controls.

**Results:**

The proportion of subjects with high levels of WBC was reduced in malaria patients as compared to the endemic control. Monocytes were diminished particularly in patients with primary malaria. The proportion of subjects with high levels of all lymphocyte subsets was decreased in all malaria groups, regardless their clinical status. Decreased proportion of subjects with high levels of CD4^+^ and CD8^+^ T-cells was found especially in the group of patients with recurrent malaria. Data analysis indicated significant increase in the proportion of the subjects with high plasmatic cytokine levels in both malaria groups, characterizing a typical cytokine storm. Recurrent malaria patients displayed the highest plasmatic IL-10 levels, that correlated directly with the CD4^+^/CD8^+^ T-cells ratio and the number of malaria episodes.

**Conclusion:**

The findings confirm that the infection by the *P. vivax* causes a decrease in peripheral blood lymphocyte subsets, which is intensified in the cases of “recurrent malaria”. The unbalanced CD4^+^/CD8^+^ T-cells ratio, as well as increased IL-10 levels were correlated with the number of recurrent malaria episodes. These results suggest that the gradual remodelling of the immune response is dependent on the repeated exposure to the parasite, which involves a strict control of the immune response mediated by the CD4^+^/CD8^+^ T-cell unbalance and exacerbated IL-10 secretion.

**Electronic supplementary material:**

The online version of this article (doi:10.1186/s12936-016-1501-5) contains supplementary material, which is available to authorized users.

## Background

*Plasmodium vivax* is widely distributed around the world, with more than 2.5 billion subjects exposed to risk of infection. *Plasmodium vivax* malaria is highly prevalent in Latin America, Asian and some Pacific regions [1]. In the past, *P. vivax* was considered the causative agent of “benign malaria” and was associated with low lethality. However, after several reports of severe forms of *P. vivax* malaria, this assumption has been challenged [2, 3]. Recent studies have shown that vivax malaria can be associated with a spectrum of severe syndrome, with risk of death similar to that observed for *Plasmodium falciparum* infection [4–9].

In Brazil, the Amazon region concentrates almost all cases of *P. vivax* infections registered countrywide, with more than 300 thousand cases per year [10]. Although the Amazon region has low transmission levels as compared to other regions in sub-Saharan Africa, the most affected population in the Amazon Basin are adults and the incidence of complications has been already reported [11, 12].

The recurrence of *P. vivax* malaria is a phenomenon that commonly occurs within a few months after the treatment of primary infection. The recurrence is defined as the reappearance of asexual forms of the parasite in the blood, up to 6 months after post-therapeutic monitoring period [13]. Usually, relapse/recrudescence may also occur for different reasons such as resistance of the parasite to the chemotherapy used [14], therapeutic failure, non-adherence to the treatment [15] and reactivation of the hypnozoites [16]. Recurrence has been rather considered a new malaria episode occurring in endemic areas after effective therapeutic intervention [17].

Although the mechanisms underlying the recurrence of are still unknown, cases of “recurrent malaria” are usually associated with impaired clinical recovery and higher morbidity, worsening the socio-economic impact of the disease [18–20]. From the epidemiologic point of view, it is likely that the recurrence favors the maintenance and transmission of the parasite, considering the early and continual presence of sexual forms of the *P. vivax* [21].

Several evidences suggest that an exacerbated inflammatory response associated to a high parasitaemia is likely to aggravate the malaria symptoms [22–27]. The exacerbated activation of the host immune system, especially T-lymphocytes is the core factor to the severe malaria pathogenicity, related to the high and out of proportion levels of the pro-inflammatory cytokines, which can be observed during the erythrocytic phase of the parasite life cycle [9, 28–30]. In general, the severity of the disease has been associated with high systemic levels of IFN-γ and TNF [31–33]. In the *P. vivax* malaria, the simultaneous increase of the TNF, IFN-γ and IL-10 has been correlated to disease progression towards a severe clinical outcome [9, 34, 35].

Counterbalancing the exacerbated inflammatory response that induces immunopathological mechanisms [28], the host immune response develops modulatory events, especially in the lymphocytic compartment [36–40]. It stands out the role of the regulatory T-cells (Treg), which may act as an important key both to the homoeostatic balance and to the control of the immunopathogenesis through the modulation of the excessive inflammatory response [41]. Treg cells are necessary to control the cellular immune response through a direct contact with the effector immune cells and by the production of regulatory cytokines including IL-10 and TGF-β [42–44]. Furthermore, anti-inflammatory cytokines, included as IL-10, have been found to regulate type 1 responses during infection during a secondary parasitic challenge in the best available mouse model for human severe malaria, demonstrating a regulatory role in the control of pathogenic responses [44].

Nevertheless little information regarding the immunological events underlying the recurrent malaria episodes are currently available. In the present study, the ex vivo phenotypic features of circulating leukocyte subsets as well as the plasmatic cytokine profile were assessed, aiming at establishing patterns of immune response characteristic of recurrent malaria.

## Methods

### Study population

This was a cross-sectional study performed during 10 months with malaria patients seeking for healthcare at the Ambulatory of the Fundação de Medicina Tropical Dr. Heitor Vieira Dourado (FMT-HVD), Manaus, Amazonas State, Brazil. The selection of malaria patients was performed by convenience, excluding individuals with chronic/degenerative diseases or pregnant women. Only those patients with positive microscopic diagnosis of *P. vivax* infection with parasitaemia higher than 500 parasite/mm^3^ were included in the study. Two sets of patients were selected for this study: patients with “primary malaria” and “recurrent malaria”. The inclusion criteria for cases of *P. vivax* “recurrent malaria” were defined as patients that presented new malaria infection within a six-month interval apart from the last episode. The occurrence of relapse/recrudescence episodes was minimized, since all patients with “recurrent malaria” underwent therapeutic regimens as recommended by the Brazilian Ministry of Health to *P. vivax* malaria (chloroquine for 3 days and primaquine for 7 or 14 days) [45–48], with strict follow-up after treatment to monitor therapeutic effectiveness, according to FMT-HVD guidelines for cure-monitoring. The periodic cure-monitoring consisted of microscopic examination of thin and thick blood smears during the first 2 months (2, 4, 7, 14, 21, 28, 40 and 60 days) after the initiation of treatment. In cases of positive microscopic results after the maximum time limit specified above, but before 6 months, the patients should be classified as new cases or “recurrent malaria”. Using this criterion, 13 patients with presumed “recurrent malaria” were excluded, as they reported presented parasitaemia levels lower than 500 parasite/mm^3^.

Fifty-five patients with *P. vivax* mono-infection [49], age ranging from 16 to 70 years, 39 males and 16 females, all presenting negative serology for dengue virus infection, were selected to compose the malaria group. Upon clinical evaluation, malaria patients were categorized into two subgroups referred as: “primary malaria” (n = 36) and “recurrent malaria” (n = 19).

Twenty healthy subjects, resident in the same geographic area was enrolled as the “endemic control” group, with age ranging 19–48 years, nine man and 11 women.

### Ethical issues and blood sampling

This study was approved by the Ethics Committee and the Superior Council at FMT-HVD (CAEE process #0044.0.114.000-11). Each participant read and signed the written informed consent form. EDTA whole blood samples (5 mL) were collected from each participant. All patients were treated according to the recommendations provided by the Brazilian Ministry of Health.

### Assessment of *Plasmodium vivax* mono-infection status

Total genomic DNA was extracted from EDTA whole blood samples, using the gDNA Blood kit (Invitrogen™, Carlsbad, CA, USA), following the instructions provided by the manufacturer. *Plasmodium vivax* mono-infection status was confirmed by Nested PCR, in the presence of specific oligonucleotides for *P. vivax*, *P. falciparum* and *Plasmodium malariae*, according to the protocol described by Snounou and Singh [50].

### Monoclonal antibody panel for immunophenotypic analyses

Anti-human cell surface/cytoplasm monoclonal antibody (mAbs) panel labeled with distinct fluorochromes were used for flow cytometric immunophenotypic analysis, including: anti-CD3-PECy7(SK7), anti-CD4-PE(RPA-T4), anti-CD8-FITC(HIT8a), anti-CD69-APC(FN50), anti-CD25-PERCP-Cy5(M-A251), anti-FoxP3-AF647(259D/C7), anti-CD19-FITC(HIB19), anti-CD5-PE(UCHT2), anti-CD16-FITC(3G8) and anti-CD56-PE(B159), all purchased from BD Bioscience, San Diego, CA, USA.

### Haematological parameters and flow cytometric analysis of lymphocyte subsets

EDTA whole blood samples were employed for assessing haematological parameters using an automated haematological analyzer (Sysmex KX-21 N^®^). An additional flow cytometric immunophenotypic analysis was also carried out as follows: briefly, 50 mL aliquots of whole blood were incubated with 5–10 mL of fluorescent mAbs for 30 min, at room temperature, in the dark. After incubation, the red blood cells were lysed with 2 mL of lysing solution (BD FACS™ Lysing Solution, BD^®^ Biosciences San Diego, CA, USA) for 10 min at room temperature, in the dark. For FoxP3 immunostaining, cells were permeabilized for 10 min at room temperature, in the dark with 2 mL of perm buffer (phosphate-buffered saline-PBS, 0.5 % saponin, 0.5 % bovine serum albumin). After one wash step with PBS, stained cells were fixed in FACS fix solution (10 g/L of paraformaldehyde, 10.2 g/L of sodium cacodylate and 6.63 g/L of sodium chloride, pH 7.2). Cells were run in a FACSCanto II^®^ flow cytometer (Becton–Dickinson Company, San Jose, CA, USA) and a total of 10,000 (100,000 for CD4/CD25/FoxP3) events collected for data analyses. Lymphocyte subsets were quantified first by specific gating strategies, using the *FlowJo software* (version 9.4.1, TreeStar Inc. Ashland, OR, USA) as represented in Additional file 1. The results were expressed initially as percentage of positive cells within the lymphocyte gate. Absolute counts for lymphocyte subsets were calculated by multiplying the percentage of gated lymphocytes obtained by flow cytometry by the absolute lymphocyte count provided from the automated haematological analyzer.

### Plasmatic cytokine quantification

The plasmatic cytokines were quantified by human Cytometric Bead Array kit for IL-2, IL-4, IL-6, IL-10, TNF and IFN-γ, all purchased from BD Biosciences Pharmingen (San Diego, CA, USA), following the instructions provided by the manufacturer. Data analysis was performed using the FCAP ArrayTM software, V.2.0 (BD Biosciences, San Jose, CA, USA). Initially, the mean fluorescence intensity (MFI) of each bead cluster was determined and forth logistic regression applied to build the standard curves. Cytokine concentrations for each sample were then extrapolated from the standard curves and data was expressed as pg/mL for each plasmatic cytokine.

### Data mining and statistical analysis

Statistical analyses were carried out using the *GraphPad Prism* software, version 5.0 (San Diego, CA, USA). Comparative analyses of continuous variables (age, haematological parameters, leukocyte subsets and plasmatic cytokines) amongst groups (endemic controls vs primary malaria vs recurrent malaria) were performed by Kruskal–Wallis followed by multiple comparisons performed by Dunn’s post-test.

The analysis of overall biomarker profile was performed by converting the original data (leukocyte subsets and plasmatic cytokine profile), obtained as continuous variables, into categorical parameters using the global median cut-off calculated for the study population. Global median values for each haematological parameter, lymphocyte subset and plasmatic cytokine were used to segregate and calculate the proportion of subjects with high biomarker levels (above the cut-off edge). The data were assembled as proposed previously by Luiza-Silva et al. [47] and Souza-Cruz et al. [48] and the biomarker profile for endemic controls used as reference curve for comparative analyses with malaria patients. The χ^2^ test was used to compare the proportions of subjects with biomarker levels above the cut-offs amongst groups.

Spearman’s test was used to sort variables that provided significant correlation. Linear regression analysis was applied to generate the best fitted line and the 95 % confidence interval bands.

In all cases, significance was considered at p < 0.05. Significance level was underscored by asterisks, as follows: (*) if p < 0.05; (**) if p < 0.005 and (***) if p < 0.0005.

## Results

### Compendium of the study population, demographic and haematological parameters

**Fig. 1:**
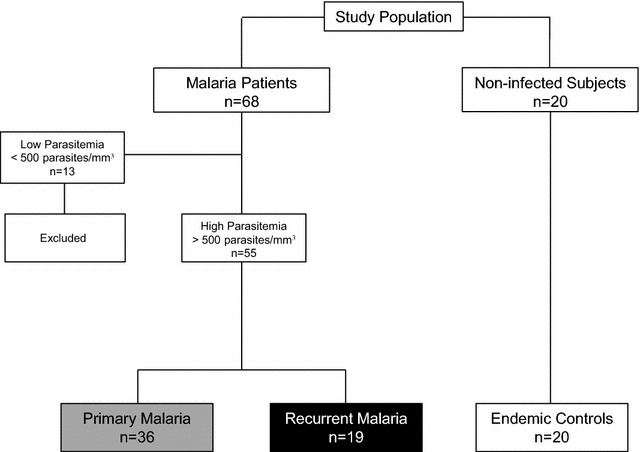
Compendium of the study population. The study enrolled malaria patients seeking for healthcare at the Ambulatory of the Fundação de Medicina Tropical Dr. Heitor Vieira Dourado (FMT-HVD), Manaus, Amazonas State, Brazil. The patient selection was performed by convenience. From the 68 patients participating in the initial inquiry, fifty-five were selected and included in the study, as patients fulfilled the inclusion criteria (parasitaemia was above 500 parasites/mm^3^). The selected patients were grouped as: primary malaria (n = 36) and recurrent malaria (n = 19) as described in “Methods” section. Twenty healthy individuals living in the same endemic area and with no history of malaria were invited to participate as endemic controls

A flowchart illustrating a compendium of the study population is shown in Fig. 1. Sixty-eight subjects were first enrolled in the present investigation due to their history of exposure to the malaria. Fifty-five patients fulfilled the inclusion criteria and were selected for the study, including 36 patients with primary malaria and 19 patients with at least one recurrent malaria episode within 6 months after post-therapeutic monitoring period. Demographic and haematological parameters of malaria patients as well as the subjects selected as endemic controls (n = 20) are presented in Table 1. Data analysis did not demonstrate any significant difference in the red blood cell (RBC) counts, haemoglobin levels and haematocrit amongst patients with malaria (whether primary or recurrent) and the endemic controls. Both malaria groups presented lower platelet numbers as compared to endemic controls (p < 0.001). The white blood cell (WBC) counts by multiple comparisons showed a reduction in both groups with malaria in relation to the endemic control individuals (p = 0.018). Malaria patients (primary and recurrent) presented lower lymphocyte counts as compared to endemic controls (p < 0.001). Patients with primary malaria showed reduced monocyte counts as compared to endemic controls (p = 0.014). No changes in the neutrophil counts were observed amongst groups (Table 1). Thrombocytopaenia and lymphopaenia are frequently noted in individuals with malaria [23, 51].

**Table 1:** Demographic and haematological parameters of the study population

	Endemic control (n = 20)	Primary malaria (n = 36)	Recurrent malaria (n = 19)	p value*
Age (years), mean (SD)	27.1 (7.4)	35.4 (14.3)	39.5 (12.0)	*0.005*
Man/woman (%)	45/55	64/36	85/15	–
Malaria episodes^a^	–	–	3.0 (1.5–4.0)	–
Last malaria attack^b^	–	–	4.0 (3.0–6.0)	–
Haematological parameters^c^				
RBC × 10^3^/mm^3^	4.8 (4.5–5.4)	4.7 (4.2–5.3)	5.0 (4.4–5.2)	0.839
Haemoglobin (g/dL)	13.2 (12.5–14.1)	13.4 (11.5–14.4)	13.1 (12.2–14.1)	0.592
Haematocrit (%)	43.9 (40.5–46.8)	41.9 (36.5–45.7)	42.0 (39–46.5)	0.461
Platelets × 10^3^/mm^3^	289.0 (212.5–325.2)	69.0 (37.8–100.5)^d^	112.0 (53–120)^d^	*<* *0.001*
WBC × 10^3^/mm^3^	6.7 (6.1–7.8)	5.7 (4.0–7.3)	5.6 (4.7–6.8)	*0.018*
Lymphocytes × 10^3^/mm^3^	2.0 (1.6–2.6)	1.3 (0.7–1.8)^d^	1.0 (0.5–1.3)^d^	*<* *0.001*
Monocytes × 10^3^/mm^3^	0.7 (0.5–0.8)	0.3 (0.2–0.5)^d^	0.5 (0.3–0.9)	*0.014*
Neutrophils × 10^3^/mm^3^	3.8 (3.5–5.1)	3.3 (2.1–5.1)	4.0 (3.0–4.7)	0.436

Patients were grouped according to their malaria diagnosis based on microscopy data

^a^The patients were tested at one time point and recurrent malaria episodes recorded

^b^Time in months

^c^Data expressed as median and interquartile range (IQR25–IQR75)

^d^Significant differences as compared to endemic controls

* Kruskal–Wallis analyses followed by Dunn’s post-test

### Biomarker profiles of primary malaria vs recurrent malaria

**Fig. 2:**
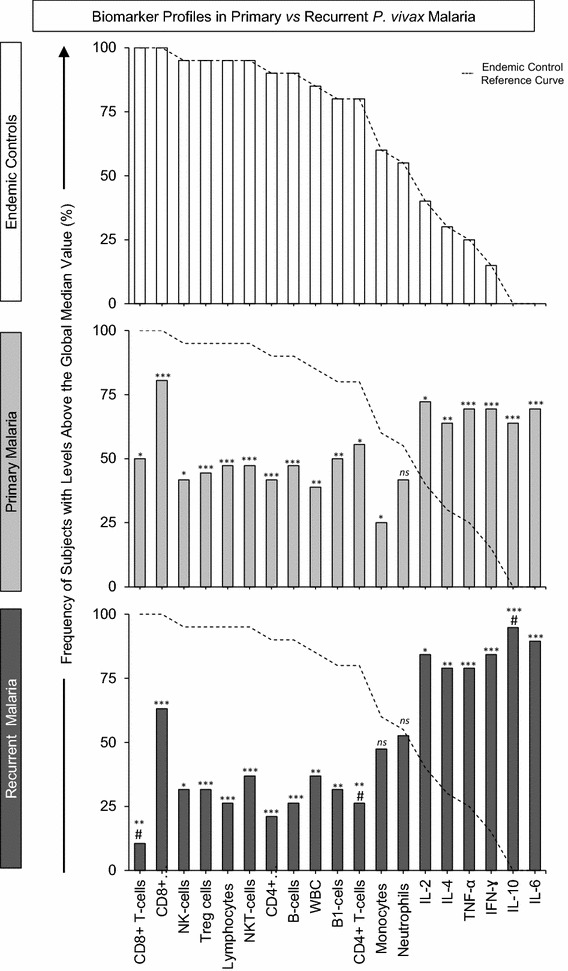
Biomarker profiles of primary malaria vs recurrent malaria. The biomarker profiles were performed by converting the original data (leukocyte subsets and plasmatic cytokine profile), obtained as continuous variables, into categorical parameters. The results were reported as the proportion of subjects with high biomarker levels above the cut-offs calculated for the study population, as described in “Methods” section. The data were arranged as descendent biomarker profile for endemic controls (*white bars*), and used as reference curve (*dotted line*) for comparative analyses with malaria patients (primary malaria = *light-gray bars* and the recurrent malaria = *dark-gray bars*). The χ^2^ test was used to identify significant differences at p < 0.05. *Asterisks* represents the significance levels (*p < 0.05, **p < 0.005 and ***p < 0.0005). Significant differences between primary and recurrent malaria were represented by *hash*

In order to compare the overall biomarker profiles for primary malaria and recurrent malaria, the continuous variables obtained originally were converted into categorical parameters using cut-off calculated for the study population. The results were reported as the proportion of subjects with high biomarker levels above the cut-offs calculated for the study population (Fig. 2). The biomarker profile for endemic controls was used as reference curve for comparative analyses with malaria patients. Data analysis showed a reduction in the proportion of subjects with high levels of WBC as compared to the endemic control reference curve. It was also observed decreased proportion of subjects with high levels of monocytes particularly in patients with primary malaria (Fig. 2). Moreover, the proportion of subjects with high levels of all lymphocyte subsets was decreased in all malaria groups, regardless their clinical status. Analyses of lymphocyte subsets showed significant decrease in the proportion of subjects with high levels of CD4^+^ T-cells (p = 0.036) and CD8^+^ T-cells (p = 0.0034), particularly in the group of patients with recurrent malaria (Fig. 2). These cell subsets have a relevant role in malaria, controlling the infection or associated with pathogenesis of severe forms of the disease, depending on their cytokine profiling [52].

The proportion of subjects with high levels of cytokines demonstrated a reversal picture characterizing a typical cytokine storm. Data analysis indicated significant increase in the proportion of the subjects with high cytokine levels in both malaria groups (Fig. 2). It was also noted an increased proportion of subjects with high plasmatic IL-10 levels, selectively in patients with recurrent malaria (p = 0.011). This was an interesting finding, since IL-10 is a well-known biomarker with regulatory role on inflammatory processes and for being directly associated to the protection in severe malaria [53]. Finally, this global analysis allowed for verifying that recurrent malaria is associated with a particular biomarker profile, characterized by decreased levels of the CD4^+^ and CD8^+^ T-cells along with a significant increase in the IL-10 production (Fig. 2).

### Ex vivo phenotypic features of circulating T-cell subsets

**Fig. 3:**
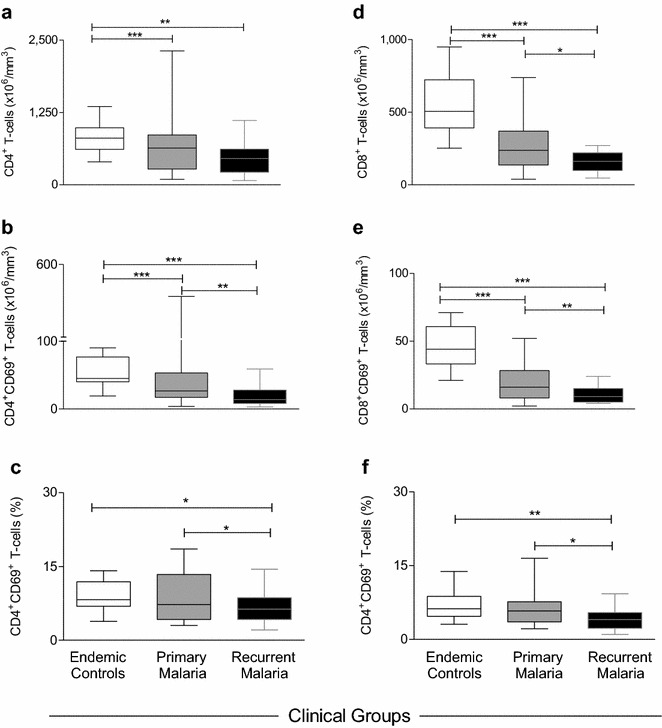
Ex vivo phenotypic features of circulating T-cell subsets. The levels and activation status of T-cell subsets were assessed in patients with primary malaria, recurrent malaria as well as endemic controls, including: **a** CD4^+^ T-cells; **b** CD4^+^CD69^+^ T-cells; **c** %CD4^+^CD69^+^ T-cells, **d** CD8^+^ T-cells; **e** CD8^+^CD69^+^ T-cells; **f** %CD8^+^CD69^+^ T-cells. Data are displayed in boxplot format (min to max, IQR25-IQR75 and median) of absolute counts (**a**, **b**, **d** and **e**) and percentage (**c** and **f**) of T-cell subsets. Multiple comparisons amongst groups were performed by Kruskal–Wallis, followed by Dunn’s post-test. Significance differences are represented by *p < 0.05; **p < 0.005 and ***p < 0.0005

The global analysis of categorical data has identified significant differences in the immune response associated with primary and recurrent malaria. Aiming at establishing useful laboratorial tools to monitor the immunological status of patients with primary and recurrent malaria, the ex vivo phenotypic features of circulating T-cell subsets were evaluated as continuous variables (Fig. 3). Data analysis confirmed that both malaria groups presented decreased absolute counts of CD4^+^ and CD8^+^ T-cells as compared to endemic controls (Fig. 3a, d). In addition, lower levels of CD8^+^ T-cells was observed in patients with recurrent malaria as compared to primary malaria (Fig. 3d). The analysis of activated T-cells demonstrated that both malaria groups presented significantly lower counts of CD4^+^CD69^+^ and CD8^+^CD69^+^ as compared to endemic controls (Fig. 3b, e). Moreover, patients with recurrent malaria displayed decreased absolute counts and percentage of CD4^+^CD69^+^ and CD8^+^CD69^+^ as compared to primary malaria (Fig. 3b, c, e, f).

### Ex vivo phenotypic features of circulating NK and NKT, Treg and B-cells

The ex vivo phenotypic features of circulating NK and NKT, Treg and B-cells, evaluated as continuous variables, are presented in Additional file 2. The results showed a decrease in NKT, Treg, B, and B1 cells in malaria patients as compared to the endemic controls, with no differences between primary and recurrent malaria (Additional file 2).

### Plasmatic cytokine profiles in primary malaria vs recurrent malaria

**Fig. 4:**
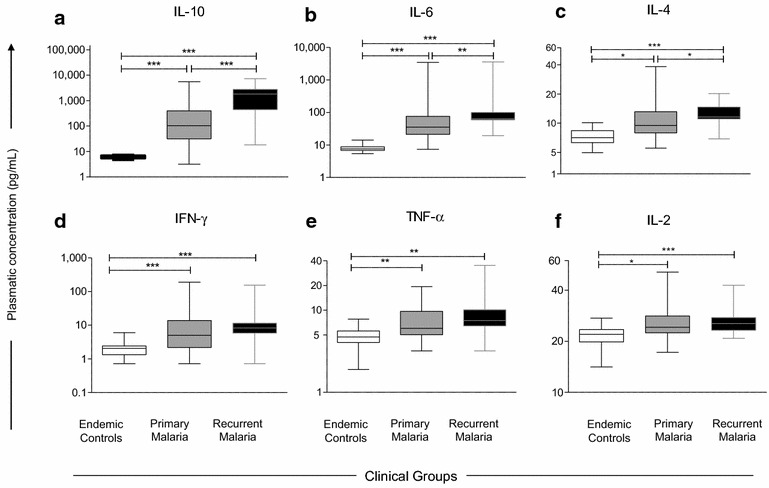
Plasmatic cytokine profiles in primary malaria vs recurrent malaria. The plasmatic cytokine levels were quantified in patients with primary malaria, recurrent malaria as well as endemic controls, using cytometric bead array as described in “Methods” section. The analysis included: **a** IL-10; **b** IL-6; **c** IL-4; **d** IFN-γ; **e** TNF-α and **f** IL-2. Data are expressed in pg/mL. Multiple comparisons amongst clinical groups were performed by Kruskal–Wallis, followed by Dunn’s post-test. Significance differences are represented by *p < 0.05; **p < 0.005 and ***p < 0.0005

The categorical analysis of plasmatic cytokine indicated that regardless their clinical status, all malaria patients presented a typical cytokine storm and also pointed out that patients with recurrent malaria group exhibited enhanced proportion of subjects with high IL-10 levels **(**Fig. 2). In order to further characterize these immunological biomarkers, the plasmatic cytokine levels were quantified as continuous variables (Fig. 4). The data analysis demonstrated that both malaria groups have significantly increased levels of IL-2, IL-4, IL-6, IL-10, TNF and IFN-γ as compared to the endemic controls. Moreover, the recurrent malaria group displayed significantly augmented levels of IL-10, IL-6, and IL-4 as compared to patients with primary malaria (p < 0.0005, p < 0.005 and p < 0.05, respectively) (Fig. 4a–c).

### Association between T-lymphocyte subsets, plasmatic IL-10 levels and number of recurrent malaria episodes

**Fig. 5:**
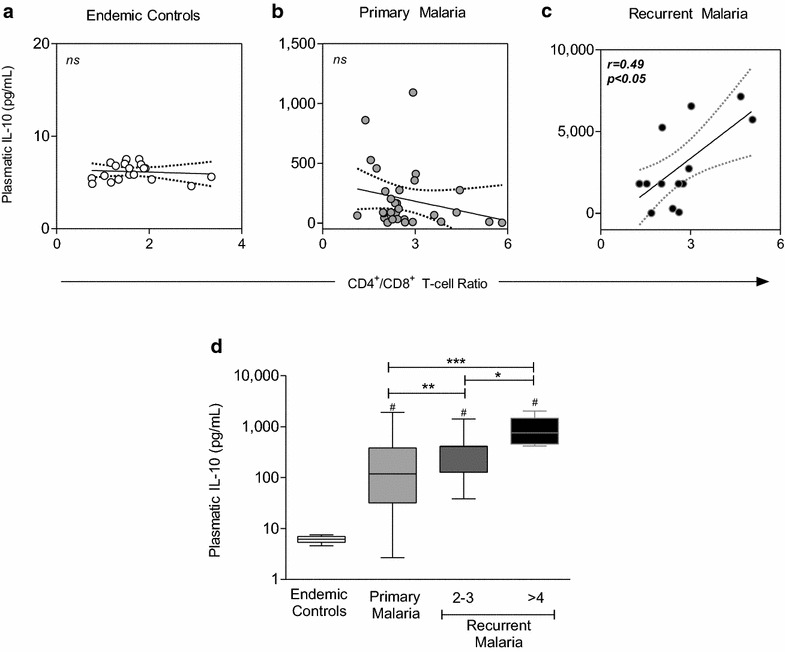
Association between T-lymphocyte subsets, plasmatic IL-10 levels and number of recurrent malaria episodes. Spearman correlation analysis was applied to identify the association between the CD4^+^/CD8^+^ T-cells (ratio) and the plasmatic IL-10 levels (pg/mL) for **a** endemic controls, **b** primary malaria and **c** recurrent malaria. Assessment of the IL-10 levels related to the previous exposition to the malaria. Additional analysis of IL-10 levels (pg/mL) in malaria patients categorized according to the number of malaria episodes (primary, 2–3 recurrent and >4 recurrent episodes) as compared to endemic controls were also carried out (**d**). Data are displayed in boxplot format (min to max, IQR25-IQR75 and median). Multiple comparisons amongst clinical groups were performed by Kruskal–Wallis, followed by Dunn’s post-test. Significance differences are represented by *p < 0.05; **p < 0.005 and ***p < 0.0005

The immune response induced by recurrent malaria revealed significant changes in the main T-cells subsets and also in relevant cytokines, particularly IL-10. Intending to verify the existence of associations amongst these variables, correlation analysis were carried out and data are presented in Fig. 5. The results demonstrated a significant positive correlation between the CD4^+^/CD8^+^ T-cells (ratio) and plasmatic IL-10 levels (pg/mL) selectively in recurrent malaria patients (Fig. 5a–c).

To add new insights to this issue, the hypothesis as to whether there is any association between IL-10 levels and the number of malaria episodes was then tested. Intergroup multiple comparisons revealed that, besides the higher IL-10 levels observed in all malaria subgroups (primary, 2–3 recurrent and >4 recurrent episodes) as compared to endemic controls, there was clear and progressive increase in the IL-10 levels according to the number of malaria episodes (Fig. 5d). Such finding reinforces the hypothesis of relevant role of the IL-10, particularly in recurrent malaria.

## Discussion

*Plasmodium vivax* is highly prevalent around the world [1]. Regardless its former association with benign malaria [3, 22], complications of *P. vivax* malaria have been reported in adult patients living in Amazon Basin endemic areas [11, 12]. It has been proposed that a balance between the pro- and anti-inflammatory responses can account for the control against the development of the severe malaria episodes [54].

Infections with *Plasmodium* ssp. are capable of inducing major changes in leukocyte profiling [9, 55–62]. In the present study, data demonstrated that patients with *P. vivax* recurrent malaria presented an unbalanced CD4^+^/CD8^+^ T-cell ratio, which was associated with a significant increase in the plasmatic IL-10 levels. Interestingly, it was verified that the IL-10 levels in patients with recurrent malaria were directly proportional to the number of malaria episodes.

In details, the ex vivo phenotypic profile of circulating leukocytes was characterized in patients with *P. vivax* primary and recurrent malaria. Relevant changes in the proportion of subjects with high levels of circulating lymphocyte subsets were found (Fig. 2). In particular, patients with recurrent malaria presented significant reduction of CD4^+^, CD8^+^ T-cells and activated T-cells (CD69-expressing CD4^+^and CD8^+^ T-cells) (Fig. 3). The reduction among those subpopulations was also evident in other studies carried out with *P. vivax* naturally infected individuals [55, 59, 63, 64]. Those authors interpreted the reduction in the lymphocyte subpopulations in the peripheral blood as a suppressant effect in the response to the *P. vivax* [55, 56, 63–65]. According to them, such reduction could be due to the high apoptosis levels or to the relocation of the cells in the liver and in other lymphoid compartments [55, 64, 66–68]. In the present study, the major changes, occurred in T-lymphocyte subsets, were observed in the group with recurrent malaria; however, further investigations must be performed to elucidate it, as the phenomenon is more accentuated in this group.

Comparison of plasmatic cytokine levels showed specific characteristics suggestive of a massive cytokine storm in patients with primary and recurrent malaria as compared to healthy endemic controls (Fig. 2). In malaria patients, the levels of IL-6 and IL-10 were increased expressively in relation to other cytokines (Fig. 4), supporting what was noted in other studies on non-complicated malaria [69–72]. It is well established that the unbalance of the pro- and anti-inflammatory mediators is a fundamental component in the malaria pathogenesis by the *P. vivax* [9, 35, 46, 58, 64, 73–76]. In this study, levels of the IL-10, IL-6 and IL-4 were higher in patients with recurrent malaria. Nonetheless, it could not be directly compared to data found in the literature due to the different methodology used. It must be mentioned that no patient participating in this study developed any complication during the acute infection. This is an important indication that within a short period of time, recurrent infections by the *P. vivax* did not increase the risk for complications, as reported previously in other studies [77, 78]. Probably, such risk is associated to the hyper-reactivity of the immune system, as it is verified in African children after recent malaria episode [79–81].

Although several studies have shown the dynamics of the subpopulations during *P. vivax* infection, the correlation between such findings and the number of malaria episodes were less explored [9, 55–62]. The findings indicate a direct association between the CD4^+^/CD8^+^ T-cells ratio and plasmatic IL-10 cytokine levels, selectively in patients with recurrent malaria (Fig. 5). Recent studies determined that after the first malaria infection, the T-cells secreting IL-10 are more stable and have a longer life span as compared to IFN-γ-producing T-cells [82–84].

Supporting these findings, the data presented here demonstrated that the levels of IL-10 were directly proportional to the number of malaria episodes [64, 83, 84]. Several reports have proposed that the resistance pattern or susceptibility to the infection by the *Plasmodium* is related to the cytokine microenvironment, with resistance to the parasite relying on cytokines such as IFN-γ, TNF, IL-12, and GM-CSF, while susceptibility is associated to the IL-10, IL-4 and TGF-β [43, 85].

The IL-10 plays an important effect inasmuch as it is capable of deactivating macrophages, and it is indirectly responsible by decreasing the production of the IFN-γ. In this way, IL-10 actuates by cushioning the potentially harmful effects of the macrophage activation on the host tissue [86]. In this study, the high production of IL-10 was remarkable in patients with recurrent malaria, being very low in patients with primary malaria. IL-10 has been indicated as an important regulator of the harmful immune responses to the host [87]. However, due to its inhibitory ability over the macrophagic hyperactivation, the secretion of such cytokine would reduce the control of the host over the circulating parasites, thus possibly favoring the relapse of the infection [85].

One of the limitations of this study was the impossibility to define which mechanisms are involved in the development of the recurrent infection, considering the multiple etiological factors involved in this phenomenon. Nevertheless, the data indicate the crucial immunoregulatory effect of the IL-10 in recurrent infections. It is also required that further investigation should be undertaken to shed light on the association between the decreased CD4^+^/CD8^+^ T-cell ratio and the increased plasmatic IL-10 levels during recurrent malaria. The findings suggest that the gradual immunity acquisition is dependent on the exposition involving a control of the immune response mediated by IL-10. Future prospective studies are required in order to assess the risk factors associated to the relapse of the *P. vivax* malaria and its relationship with different components of the immune response.

## Conclusion

Although this study has been conducted with a relatively small number of patients, our findings have shown that recurrent malaria, within 6 months after the end of therapeutic monitoring period, induces a relevant IL-10-mediated response, suggesting the occurrence of a gradual acquisition of modulatory immunity. The present study is a descriptive investigation and does not report the mechanisms underlying the development of distinct patterns of immune response in patients with recurrent malaria. The hypothesis that IL-10 would have a protective role against complications of recurrent malaria still requires further investigation. It is important to keep in mind that, due to its modulatory activity, the high levels of the IL-10 would also favor the occurrence of recrudescence/relapse of *P. vivax* malaria.

## Additional files

10.1186/s12936-016-1501-5 Representative flow cytometric analysis of lymphocyte subsets. Peripheral blood lymphocytes were first selected based on their morphometric features (size/FSC forward scatter and granularity/SSC side scatter) on pseudocolour plot (A). Following, the phenotypic features were evaluated to quantify cell-substs and the activation status, including, CD4^+^ and CD8^+^ T-cells (B and C); CD69^+^CD4^+^ and CD69^+^CD8^+^ T-cells (D and E); CD56^+^CD16^+^ within CD3^−^ events - NK-cells (F), CD3^+^CD56^+^ NKT-cell (G) FoxP3^+^CD25^+^CD4^+^ within CD4^+^ events-Treg cells (H) and CD19^+^ B-cells, and CD19^+^CD5^+^ B1-cells (I). All analysis were performed using the *FlowJo software* (version 9.4.1, TreeStar Inc. Ashland, OR, USA)
10.1186/s12936-016-1501-5 Ex vivo phenotypic features of circulating NK and NKT, Treg and B-cells. The levels lymphocyte subsets were assessed in patients with primary malaria, recurrent malaria as well as endemic controls, including: A) NK-cells; B) NKT-cells; C) Treg-cells; D) B-cells; and E) B1-cells. Data are displayed in boxplot format (min to max, IQR25-IQR75 and median). Multiple comparisons amongst clinical groups were performed by Kruskal–Wallis, followed by Dunn’s post-test. Significance differences are represented by * for p < 0.05; ** for p < 0.005 and *** for p < 0.0005
