# Supplementary material for: Immune response pattern in recurrent Plasmodium vivax malaria
Source: Malar J. 2016 Aug 31;15(1):445. doi: 10.1186/s12936-016-1501-5 (PMC5007810; doi:10.1186/s12936-016-1501-5)
Supplement: Supplementary file 1 — 10.1186/s12936-016-1501-5 Representative flow cytometric analysis of lymphocyte subsets. Peripheral blood lymphocytes were first selected based on their morphometric features (size/FSC forward scatter and granularity/SSC side scatter) on pseudocolour plot (A). Following, the phenotypic features were evaluated to quantify cell-substs and the activation status, including, CD4+ and CD8+ T-cells (B and C); CD69+CD4+ and CD69+CD8+ T-cells (D and E); CD56+CD16+ within CD3− events - NK-cells (F), CD3+CD56+ NKT-cell (G) FoxP3+CD25+CD4+ within CD4+ events-Treg cells (H) and CD19+ B-cells, and CD19+CD5+ B1-cells (I). All analysis were performed using the FlowJo software (version 9.4.1, TreeStar Inc. Ashland, OR, USA) [file 12936_2016_1501_MOESM1_ESM.pptx]

## Slide 1
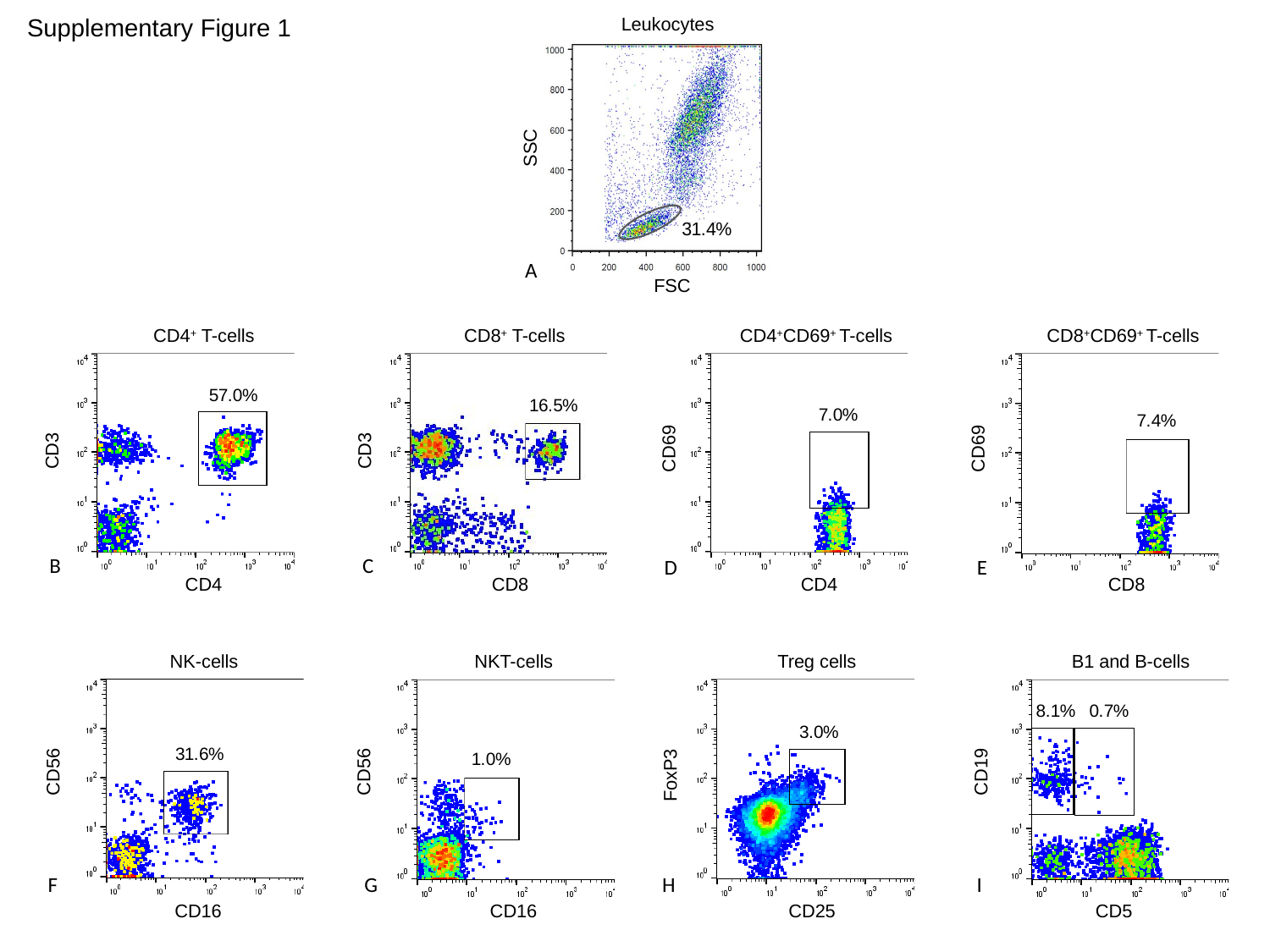

Supplementary Figure 1
Leukocytes
SSC
A
FSC
CD4+ T-cells
CD8+ T-cells
CD4+CD69+ T-cells
CD8+CD69+ T-cells
CD69
CD69
CD3
CD3
B
C
D
E
CD4
CD8
CD4
CD8
NK-cells
NKT-cells
Treg cells
B1 and B-cells
FoxP3
CD56
CD56
CD19
F
G
H
I
CD16
CD16
CD25
CD5
