# Supplementary material for: Immune response pattern in recurrent Plasmodium vivax malaria
Source: Malar J. 2016 Aug 31;15(1):445. doi: 10.1186/s12936-016-1501-5 (PMC5007810; doi:10.1186/s12936-016-1501-5)
Supplement: Supplementary file 2 — 10.1186/s12936-016-1501-5 Ex vivo phenotypic features of circulating NK and NKT, Treg and B-cells. The levels lymphocyte subsets were assessed in patients with primary malaria, recurrent malaria as well as endemic controls, including: A) NK-cells; B) NKT-cells; C) Treg-cells; D) B-cells; and E) B1-cells. Data are displayed in boxplot format (min to max, IQR25-IQR75 and median). Multiple comparisons amongst clinical groups were performed by Kruskal–Wallis, followed by Dunn’s post-test. Significance differences are represented by * for p < 0.05; ** for p < 0.005 and *** for p < 0.0005 [file 12936_2016_1501_MOESM2_ESM.pptx]

## Slide 1
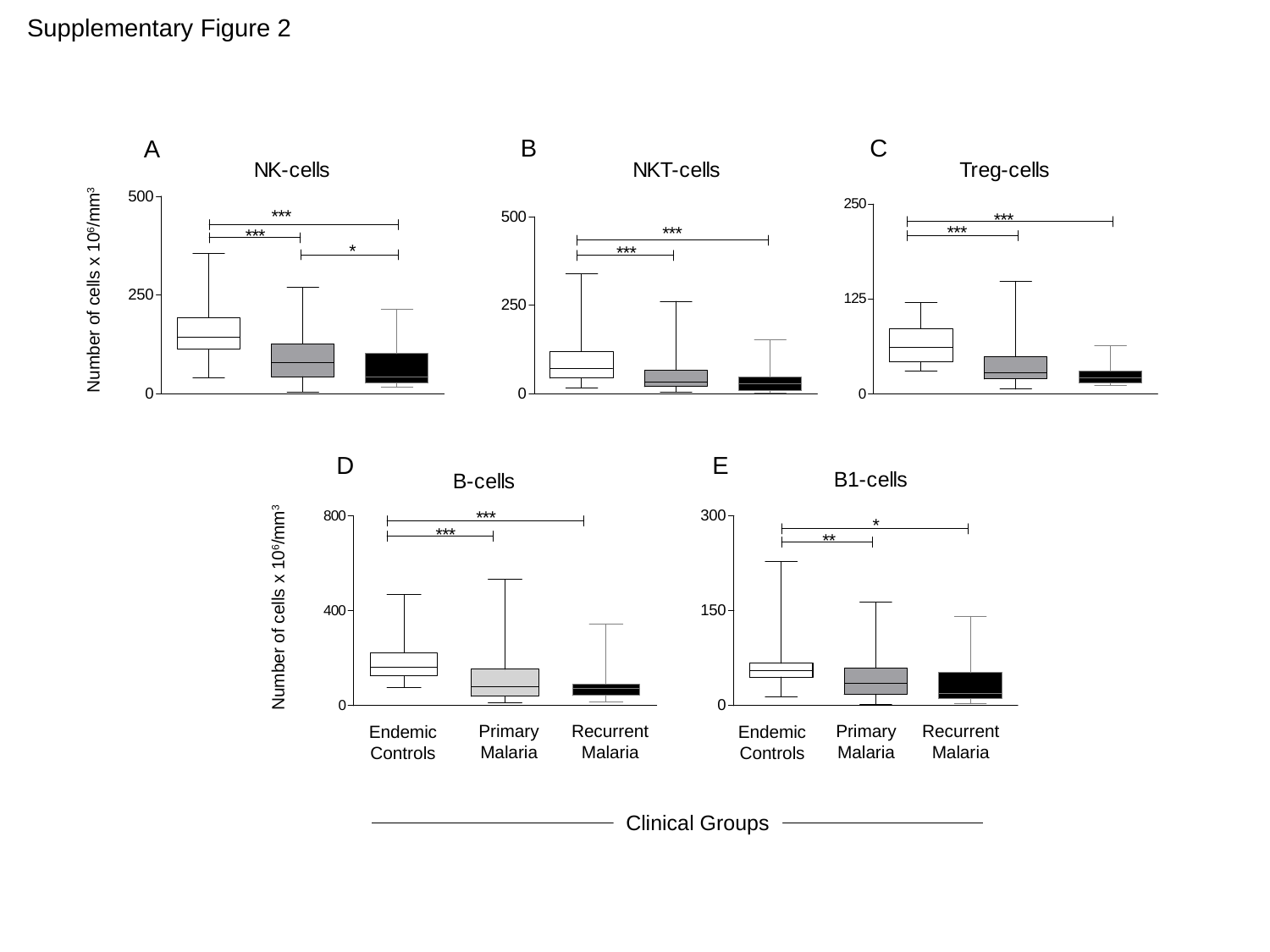

Supplementary Figure 2
Number of cells x 106/mm3
Number of cells x 106/mm3
Endemic Controls
Primary Malaria
Recurrent Malaria
Endemic Controls
Primary Malaria
Recurrent Malaria
Clinical Groups
